# The Emerging Factors and Treatment Options for NAFLD-Related Hepatocellular Carcinoma

**DOI:** 10.3390/cancers13153740

**Published:** 2021-07-26

**Authors:** Chunye Zhang, Ming Yang

**Affiliations:** 1 Department of Veterinary Pathobiology, University of Missouri, Columbia, MO 65211, USA; czvw9@mail.missouri.edu; 2 Department of Surgery, University of Missouri, Columbia, MO 65211, USA

**Keywords:** hepatocellular carcinoma, nonalcoholic fatty liver diseases, genetic and epigenetic factors, transcriptional and post-transcriptional factors, diagnosis, treatment

## Abstract

**Simple Summary:**

Nonalcoholic fatty liver disease (NAFLD) is the most common chronic liver disease, and it is an increasing factor in the cause of hepatocellular carcinoma (HCC). The incidence of NAFLD has increased in recent decades, accompanied by an increase in the prevalence of other metabolic diseases, such as obesity and type 2 diabetes. However, current treatment options are limited. Both genetic factors and non-genetic factors impact the initiation and progression of NAFLD-related HCC. The early diagnosis of liver cancer predicts curative treatment and longer survival. Some key molecules play pivotal roles in the initiation and progression of NAFLD-related HCC, which can be targeted to impede HCC development. In this review, we summarize some key factors and important molecules in NAFLD-related HCC development, the latest progress in HCC diagnosis and treatment options, and some current clinical trials for NAFLD treatment.

**Abstract:**

Hepatocellular carcinoma (HCC) is the most common type of primary liver cancer, followed by cholangiocarcinoma (CCA). HCC is the third most common cause of cancer death worldwide, and its incidence is rising, associated with an increased prevalence of obesity and nonalcoholic fatty liver disease (NAFLD). However, current treatment options are limited. Genetic factors and epigenetic factors, influenced by age and environment, significantly impact the initiation and progression of NAFLD-related HCC. In addition, both transcriptional factors and post-transcriptional modification are critically important for the development of HCC in the fatty liver under inflammatory and fibrotic conditions. The early diagnosis of liver cancer predicts curative treatment and longer survival. However, clinical HCC cases are commonly found in a very late stage due to the asymptomatic nature of the early stage of NAFLD-related HCC. The development of diagnostic methods and novel biomarkers, as well as the combined evaluation algorithm and artificial intelligence, support the early and precise diagnosis of NAFLD-related HCC, and timely monitoring during its progression. Treatment options for HCC and NAFLD-related HCC include immunotherapy, CAR T cell therapy, peptide treatment, bariatric surgery, anti-fibrotic treatment, and so on. Overall, the incidence of NAFLD-related HCC is increasing, and a better understanding of the underlying mechanism implicated in the progression of NAFLD-related HCC is essential for improving treatment and prognosis.

## 1. Introduction

Primary liver cancer was the sixth most commonly diagnosed and the third most common cause of cancer-related death worldwide in 2020 [1]. Hepatocellular carcinoma (HCC) comprises approximate 80% of primary liver cancer (PLC) cases [2], whereas cholangiocarcinoma (CCA) represents 10% to 15% of PLC cases [1]. Combined hepatocellular-cholangiocarcinoma (CHC) is a rare case in PLC [3,4]. Multiple factors contribute to the development of HCC, such as diet [5,6], infection with hepatitis viruses [7,8], alcohol abuse [9,10], and bioactive compounds [11,12]. Recent studies show that hepatitis C viral infection is the most common causal factor for HCC but that it shows a declining trend, whereas nonalcoholic fatty liver disease (NAFLD) or its advanced subtype, nonalcoholic steatohepatitis (NASH), is the most rapidly growing factor contributing to HCC development in the United States [13]. Myers et al. reported that in a study performed in western Switzerland, NAFLD or metabolic-associated fatty liver disease (MAFLD) was found to be an increased inducing factor for HCC incidence, being especially higher in women than in men, whereas other etiologies remained stable [14]. The early diagnosis of liver cancer is critically important for curative treatment, since early-stage HCC can be locally ablated or resected. Surgery such as laparoscopic surgery is recommended as the first-line therapy for HCC patients with an early diagnosis [15]. Unfortunately, NAFLD-related HCC progression does not have obvious clinical symptoms, which means that most cases are found in the late stage of the disease [16]. Furthermore, the increasing prevalence of NAFLD worldwide and limited therapeutic options may raise the incidence of HCC [17].

**Figure 1:**
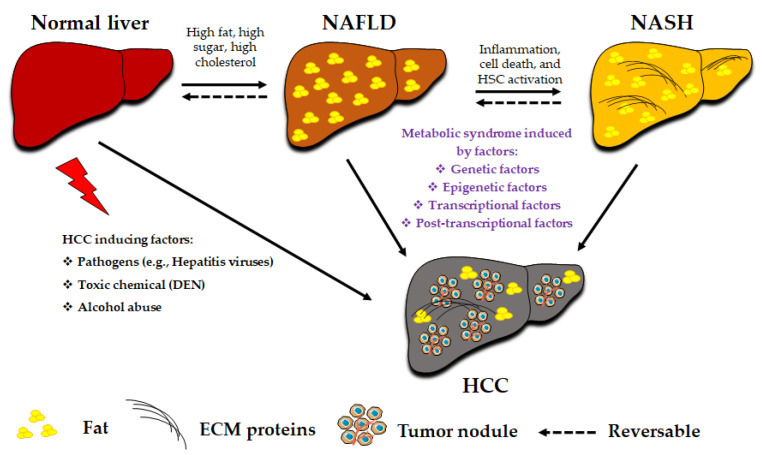
Factors causing the development and progression of NAFLD-related HCC and other etiologies causing HCC aside from NAFLD. Abbreviations: DEN: diethylnitrosamine; ECM: extracellular matrix proteins; HCC: hepatocellular carcinoma; HSC: hepatic stellate cell; NAFLD: nonalcoholic fatty liver disease; NASH: nonalcoholic steatohepatitis.

The development of HCC is associated with age, sex, geography, and etiology [2]. Men have a much higher chance of developing HCC than women. Furthermore, the diagnosis of HCC in men aged ≥60 years has dramatically increased, and milder changes have been observed in women. The sex-induced difference in the incidence of HCC is dependent not only on the hormone estrogen [18], but also on other factors, such as gut microbiota, bile acids (BAs), and microRNAs (miRNAs) [19]. Factors causing the development of NAFLD-related HCC (Figure 1), including epigenetic and genetic factors and transcriptional and post-transcriptional factors, as well as their diagnosis and treatment, are discussed in this study.

## 2. Genetic Factors

Genetic factors, such as patatin-like phospholipase domain-containing protein 3 (*PNPLA3*) [20,21], transmembrane-6 superfamily member 2 (*TM6SF2*) [22], and programmed cell death-1 (*PDCD1*) encoding PD-1, are associated with NAFLD-related HCC initiation and progression [23].

The allele alteration of rs7421861 A > G in *PDCD1* gene is associated with a decreased frequency of NAFLD-HCC progression, since the wild-type A allele has been observed more in patients with NAFLD-HCC [23]. In contrast, the allele alteration of rs10204525 C > T in *PDCD1* gene increased the progression of NAFLD-HCC compared to the wild-type C allele. In silico analysis showed that the rs7421861 A allele in *PDCD1* gene was associated with the higher expression of PD-1 compared to the G allele, which suggests that the G allele decreases PD-1-mediated immune exhaustion to suppress HCC growth [23]. In addition, a mutation of the rs7421861 allele in *PDCD1* gene, located in intron 1 with richness in regulatory and splicing sites, may cause splicing disruption, translational inhibition, and a change in the mRNA secondary structure [24].

Patients with the rs58542926 C > T genetic variant of the *TM6SF2* gene, encoding the E167K amino acid substitution, showed a lower serum lipid content, but had more severe hepatic steatosis, inflammation, ballooning, and fibrosis, and were more susceptible to develop NASH [25,26]. The T allele was shown to be associated with the reduction of *TM6SF2* gene and protein expression in the liver [26]. A meta-analysis study showed that the rs58542926 T allele in *TM6SF2* gene had a significant association with HCC development compared to the C allele [27]. This effect may be mediated by regulating the cell cycle [28] and upregulating inflammatory cytokines, such as IL-2 and IL-6 [29].

The rs599839 A > G variant, localized in the genetic cluster of cadherin EGF LAG seven-pass G-type receptor 2 (*CELSR2*)-proline/serine-rich coiled-coil protein 1 (*PSRC1*)- sortilin 1 (*SORT1*), was associated with reduced severity of dyslipidemia in NAFLD patients with a higher risk of cardiovascular comorbidities [30]. This variant was associated with increased hepatic expression of CELSR2, PSRC1, and SORT1 in NAFLD patients. In addition, the data from The Cancer Genome Atlas (TCGA) showed that PSRC1-overexpression promoted HCC development [30]. However, this variant was not significantly correlated with hepatic steatosis, ballooning, lobular inflammation, or fibrosis.

In addition, the rs641738 C > T variant, near two genes encoding membrane-bound O-acyltransferase domain-containing 7 (MBOAT7) and transmembrane channel-like 4 (TMC4), was shown to be associated with the progression of NAFLD and liver fibrosis [31]. The loss of function of MBOAT7 is identified to be a factor contributing to NAFLD progression. Another study also reported that the rs641738 T allele of *MBOAT7* gene is associated with NAFLD-related HCC in non-cirrhotic patients [32]. However, the rs641738 C > T variant in *TMC4* gene was not found to be a genetic risk in relation to increasing the development of NAFLD [33]. Similarly, in this study, the protein level of MBOAT7 was found to be lower in the liver of NAFLD patients. The rs641738 C allele has been shown to be associated with a high expression of MBOAT7 that localizes into the membranes, which helps deliver membrane metabolites into intracellular compartments. In contrast, the T risk allele is associated with the reduction of MBOAT7, which is favorable for the increase of saturated phospholipids and triglyceride (TG) synthesis [34]. More evidence is needed to support the role of the rs641738 variant of *MBOAT7/TMC4* in the susceptibility of NAFLD and NAFLD-related HCC progression.

## 3. Epigenetic Factors

Instead of a change in DNA sequence, epigenetic changes modulated by factors such as age and environment can also impact the progression of NAFLD-related HCC. Epigenetic factors, including DNA methylation, long non-coding RNAs (lncRNAs), and miRNAs, are considered to have profound effects on NAFLD-related HCC progression.

### 3.1. DNA Methylation

DNA methylation is implicated in liver fibrosis, cirrhosis, and HCC. Hypermethylation of CpG islands in genes such as *CELSR1* and collapsin response mediator protein 1 (*CRMP1*), and hypomethylation of CpG loci in small proline-rich protein 3 (*SPRR3*) and tumor necrosis factor ligand superfamily member 15 (*TNFSF15*) genes were found in HCC and cirrhotic liver tissues compared to noncirrhotic control liver tissues [35]. Hypermethylation in promoters of genes such as Ras association domain-containing protein 1 (*RASSF1A*) and docking protein 1 (*DOK1*) was associated with the pathogenesis of hepatocarcinogenesis [36]. DNA methylation at specific CpGs within genes known to affect fibrogenesis, such as peroxisome proliferator-activated receptor alpha (*PPARα*), transforming growth factor-beta 1 (*TGF-β1*), and platelet-derived growth factor alpha (*PDGFα*) genes, was observed in patients with NAFLD or alcoholic liver disease (ALD) associated with the progression of fibrosis [37]. In addition to the dysregulation of DNA methylation, histone acetylation or methylation-mediated epigenetic changes can lead to cell apoptosis in the development of NAFLD and HCC. This specific subject has been well-reviewed in another published paper [38], and is thus not discussed in this paper.

### 3.2. Long Non-Coding RNAs

LncRNAs, defined as RNAs with a length of ≥200 nucleotides that are not translated into functional proteins, play an important role in endoplasmic reticulum (ER) stress and oxidative stress. The expression of more than 3000 lncRNAs was observed to be changed in the liver tissues of db/db mice fed with a NASH diet, and the expression of 381 lncRNAs was significantly increased during NAFLD progression to NASH [39]. Among these, LncRNA gm9795 can upregulate ER stress molecules and the nuclear factor kappa B (*NF-κB*)/c-Jun N-terminal kinase (*JNK*) signaling pathway to increase proinflammatory cytokine production, such as that of TNF-α, interleukin-6 (IL-6), and IL-1β. Increased expression of LncRNA *SNHG20* was observed in the livers of NAFLD-related HCC-bearing mice and human patients with NALFD-related HCC [40]. Silencing *SNHG20* can delay the progression of NAFLD to HCC [40]. In addition, lncRNAs (e.g., *MYLK-AS1*) can act as competitive endogenous RNA, inducing miRNAs (e.g., *miR-424-5*) to regulate tumor angiogenesis in HCC [41]. 

### 3.3. MicroRNAs

MiRNAs contribute to the progression of NAFLD, liver fibrosis, and HCC development. For example, microRNA-21 (*miR-21*) has been shown to impair lipid metabolism in mice with NAFLD and human liver cancer cell line HepG2 cells, and to inhibit the progression of xenograft tumors induced by HepG2 cells, as *miR-21* knockdown can impair lipid accumulation and tumor growth by targeting HMG-Box transcription factor 1 (*HBP1*)-*p53*, part of the sterol regulatory element-binding protein 1c (*SREBP1c*) signaling pathway [42]. In addition, hepatic *miR-21* expression has been shown to be upregulated in a methionine-choline-deficient (MCD) diet-induced mouse NASH model and in human patients with NASH [43]. Suppressing *miR-21* function with antagomir-21 can reduce liver injury, inflammation, and fibrosis in low-density lipoprotein (LDL) receptor-deficient mice, but not in *PPARα*-deficient mice [43]. A recent study using a doxycycline-inducible transgenic zebrafish model (LmiR21) with hepatic overexpression of *miR-21* showed that *miR-21* overexpression contributed to liver steatosis, inflammation, and fibrosis, the broad spectrum of NAFLD [44]. Moreover, LmiR21 zebrafishes showed the NAFLD-HCC phenotype at 10 months post-fertilization, and they also showed a higher percentage of chemical-induced liver fibrosis and HCC compared to wild-types under the chemical stimuli.

Hepatocyte-specific *miR-122a* accounts for 70% of the total miRNAs in the liver and is downregulated in about 70% of human HCC and all HCC-derived cell lines [45]. *MiR-122a*-deficient mice develop reversible steatohepatitis, fibrosis, and HCC [46]. In addition, the incidence of HCC shows a sexual disparity, being 3.9 times higher in male mice compared to female mice.

The expression of *miR-223* in hepatocytes is highly increased in mice when feeding on a high-fat diet (HFD) and in the liver samples of patients with NASH. Feeding with an HFD significantly enhanced the expression of proinflammatory and cancer-related genes in *miR-233*-knockout mice compared to that in wild-type mice [47].

In addition, some chemical RNA modifications are emerging factors of epigenetic regulation. For example, N6-methyladenosine (m^6^A), the most abundant chemical modification of eukaryotic mRNA, plays a critical role in regulating adipogenesis [48]. Methyltransferase-like 3 (METTL3)-mediated m^6^A modification can inhibit the suppressor of cytokine signaling 2 (SOCS2) to promote HCC progression [49].

## 4. Transcriptional Factors

**Figure 2:**
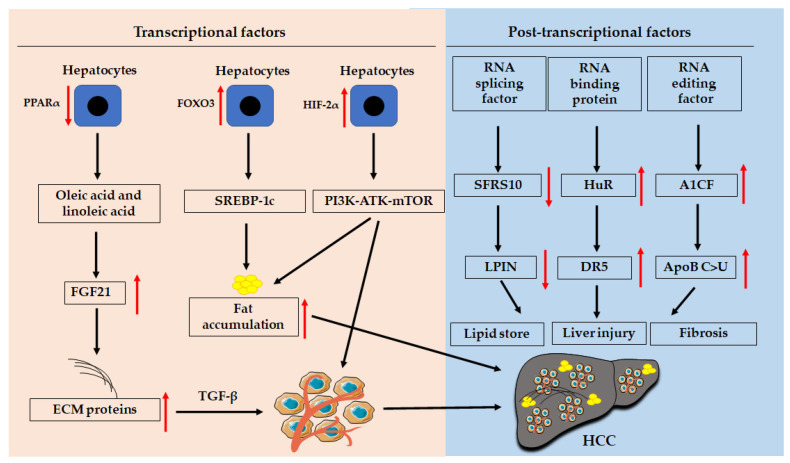
Transcriptional and post-transcriptional factors mediated the progression of NAFLD and HCC. Abbreviations: AKT: protein kinase B; ApoB: apolipoprotein B; A1CF: catalytic polypeptide 1 complementation factor; DR5: death receptor 5; FOXO: Forkhead box; HIF-2α: hypoxia-inducible factor-2 alpha; HuR: human antigen R; LPIN: lipin; mTOR: mechanistic target of rapamycin; PI3K: phosphoinositide 3-kinase; PPARs: peroxisome prolifera-tor-activated receptors; SFR10: splicing factor, arginine/serine-rich 10; SREBP-1: sterol regulatory element-binding transcription factor-1; TGF-β: transforming growth factor-beta.

Transcriptional factors, such as E2Fs transcriptional factors, hypoxia-inducible factors (HIFs), Forkhead box (FOXO), and PPARs, modulate NAFLD progression through different signaling pathways (Figure 2). For example, PPARγ can regulate lipid metabolism by regulating the Toll-like receptor 4 (*TLR4*)/*NF-κB* signaling pathway [48].

### 4.1. E2F1 and E2F2

The expression of transcription factors E2F1 and E2F2 is positively correlated and increased in NAFLD-related HCC. Their deficiency decreased hepatocarcinogenesis induced by HFD plus diethylnitrosamine (DEN) administration, with a reduction of lipid accumulation [50]. The molecular mechanism shows that E2F1 reversely modulates carnitine palmitoyltransferase 2 (CPT2), an essential enzyme for fatty acid oxidation, the downregulation of which promotes HCC development via acylcarnitine accumulation in a lipid-rich environment [51]. E2F1 is upstream of the transcription factor of ribosome binding protein 1 (RRBP1), which can be upregulated by high glucose. Inhibiting *E2F1* expression decreased the expression of RRBP1, remarkedly reducing the proliferation and metastasis of HepG2 cells [52].

### 4.2. FOXOs

FOXO transcriptional factors play important roles in regulating hepatic glucose [53] and lipid homeostasis [54], cell growth and apoptosis [55], and liver inflammation and fibrosis [56]. FOXOs are the downstream signaling of protein kinase B (AKT), which can phosphorylate the serine or threonine of FOXOs to regulate multiple cellular functions. FOXO3 can activate the promoter of SREBP1c to aggravate liver TG and intrahepatic lipid accumulation [54]. However, feeding with an HFD induced more severe hepatic steatosis and fibrogenesis in *Foxo1/3/4* triple knockout mice compared to wild-type mice via upregulating profibrotic genes such as C-C motif chemokine ligand 2 (*CCL2*), alpha-1 type I collagen (*Col1A1*), and *TGF-β* [56].

### 4.3. HIFs

HIFs, such as HIF-1α and HIF-2α, are transcription factors induced in response to a hypoxic environment, which plays a pivotal role in liver inflammation [57] and tumor growth [58]. Hypoxia also affects NAFLD-HCC progression, since HIF-2α was found to be increased in HCC tissues from NAFLD-HCC patients compared to tissues from non-NAFLD-HCC subjects [59]. The upregulation of HIF-2α was negatively associated with the overall survival (OS) of HCC patients and was positively associated with hepatic lipid accumulation and activation of the phosphoinositide 3-kinase (*PI3K*)*-AKT*-mechanistic target of rapamycin (*mTOR*) signaling pathway [59].

### 4.4. KLF6

As a transcription factor, Krüppel-like factor 6 (KLF6) plays essential roles in cellular processes, including cell proliferation, differentiation, and cell death [60]. In NAFLD, KLF6 regulates liver glucose and lipid metabolism by regulating the activity of *PPARα* and *PPARα*-regulated genes such as phosphoenolpyruvate carboxykinase (*PEPCK*) [61]. In addition, KLF6 binds the promoter of glucokinase (GCK) in NAFLD, which can regulate insulin resistance and the glucose level in the blood [62]. A mutation of a polymorphism, KLF6 intervening sequence (IVS) 1–27 G > A, was found to be positively associated with liver fibrosis in NAFLD patients [63]. Accumulating studies show that *KLF6* is a tumor suppresser gene against HCC [64,65,66].

### 4.5. PPARs

PPARs are important transcriptional factors in modulating liver inflammation [67], lipid metabolism [68], and cancer growth [69]. All three PPAR subtypes, including PPARα [70], PPARβ/δ [71], and PPARγ [72], play important roles in lipid metabolism, either in the liver or adipose tissues. For example, hepatocyte-specific *PPARα* deficiency mice showed a significant increase in oleic acid and linoleic acid compared with wild-type mice in fasting, partly due to a fasting-induced increase in fibroblast growth factor 21 (FGF21) expression [73]. Metabolic syndrome, such as insulin resistance and hepatic steatosis, can be induced by activating JNK to suppress PPARα function. However, long-term *JNK* deficiency can result in CCA by interrupting cholesterol metabolism and bile acid homeostasis [74]. In contrast, metabolites such as 3-hydroxybutyric acid, induced by treatment with the antiangiogenic agent apatinib, can induce PPARα activation in the liver tissue to inhibit tumor growth [75].

Additionally, PPARβ/δ [76] and PPARγ [77] are implicated in liver homeostasis by regulating glucose and fatty acid metabolism. PPAR-γ agonists (e.g., pioglitazone) show clinical effects in the reduction of hepatic or visceral fat and necroinflammation in human patients [78]. PPARβ/δ activator GW501516 can prevent HFD-induced hypertriglyceridemia and hepatic fatty acid oxidation, and increase the production of 16:0/18:1-phosphatidylcholine, an endogenous ligand for PPARα in the liver [79]. A bioinformatic study showed that PPARγ was overexpressed in the livers of human patients with HCC, which was associated with poor OS [80]. However, the exact roles of PPARs in the progression of NAFLD-related HCC need to be illustrated.

### 4.6. SREBP-1

SREBP-1 is a transcriptional factor and plays a pivotal role in the proliferation and metastasis of liver cancer cells by regulating fatty acid synthesis and [81] and suppressing liver inflammation [82]. Other factors, such as long-chain acyl CoA synthetase 4 (ACSL4) [83], caveolin-1 (Cav1) [84], and zinc fingers and homeoboxes 2 (ZHX2) [85], can regulate lipid metabolism via the *SREBP1* signaling pathway. Lipid metabolism is correlated tightly with glucose metabolism in HCC. Inhibition of SREBP-1 expression can suppress glucose metabolism in HCC cells, resulting in a synergistic anti-tumor effect combined with immunotherapy with Sorafenib on HCC in vivo [81].

Furthermore, there are some other transcriptional factors implicated in NAFLD-related HCC in murine models and clinical studies, such as apoptosis antagonizing transcription factor (AATF) [86,87] and carbohydrate responsive element-binding protein (ChREBP) [88,89].

## 5. Post-Transcriptional Modification

Post-transcriptional factors (Figure 2) such as RNA-binding proteins (RBPs) and RNA splicing factor (SF) contribute to liver damage, NAFLD development, and HCC progression [90]. Novel anti-HCC therapies can be developed based on post-transcriptional regulation, such as the administration of adenovirus-mediated trans-splicing ribozymes [91,92]. The underlying mechanism of post-transcriptional modification in NAFLD-related HCC progression needs to be investigated further.

### 5.1. RNA Splicing Factor

Dysregulation of RNA splicing factors contributes to the development of steatosis and NAFLD progression [93]. Silencing of some splicing machinery components in vitro, such as RNA binding motif protein 45 (RBM45) and staphylococcal nuclease domain containing 1 (SND1), can inhibit fat accumulation by modulating the expression of key de novo lipogenesis enzymes [93]. Dysregulation of RBM45 and SND1 has been associated with the progression of cancers, including HCC [94]. A splicing factor, arginine/serine-rich 10 (SFRS10) in the liver, directly regulates the splicing of lipin 1 encoded by the *LPIN* gene, a key regulator of lipid metabolism. SFRS10 has been shown to be downregulated in obese human livers and livers in HFD-fed mice [95]. Reducing or inhibiting SFRS10 expression can increase lipid accumulation in hepatocytes and plasma TG and very-low-density lipoprotein (VLDL) secretion by increasing the lipogenic β isoform of LPIN1 [95].

### 5.2. RNA-Binding Proteins

Sirtuin 1, encoded by the *SIRT1* gene, can deacetylate an RNA-binding protein quaking 5 (QKI 5), which inhibits TG synthesis in vivo and in vitro via the *PPARα/FoxO1* signaling pathway and suppresses NAFLD progression in mice [96]. Another study showed that a broadly expressed RNA-binding protein human antigen R (HuR) can accelerate NASH progression by increasing death receptor 5 (DR5)/caspase 8/caspase 3-mediated hepatocyte death and liver injury [97].

Aberrant expression of RBPs has been shown across many malignant tumors, including HCC [98,99]. A high score based on the expression of these RBPs was associated with poor overall survival of HCC [99]. Another study showed that aberrant expression of four key *RBPs*, including mitochondrial ribosomal protein L54 (MRPL54), enhancer of zeste homolog 2 (EZH2), PPARγ coactivator 1 alpha (PPARGC1A), and eukaryotic translation initiation factor 2-alpha kinase 4 (EIF2AK4), can be applied to HCC prognosis [100].

### 5.3. RNA Editing

The inactivation or low activity of apolipoprotein B (ApoB) is associated with poor prognosis of HCC, upregulation of oncogenic and metastatic factors, and the inhibition of tumor suppressor genes [101,102], such as *p53* and phosphatase and tensin homolog (*PTEN*). The *ApoB* mRNA editing enzyme, catalytic polypeptide 1 (APOBEC1) complementation factor (A1CF) regulates posttranscriptional *ApoB* mRNA editing (C > U). Aged hepatocyte-specific *A1CF*-transgenic mice can spontaneously develop hepatic fibrosis and HCC, and disease can be accelerated when those mice are fed a high-fructose high-fat diet [103]. In addition, the expression of A1CF was associated with advanced fibrosis and low survival in NAFLD-related HCC patients [103].

Overall, both genetic and epigenetic factors play critical roles in NAFLD-related HCC progression. Furthermore, recent studies show that NAFLD/NASH modulates intrahepatic immune responses to inhibit anti-tumor immunity, resulting in the progression of HCC. For example, Tim Greten et al. reported that dysregulation of lipid metabolism, specifically for linoleic acid, causes more oxidative damage in mitochondria during NAFLD, resulting in a dramatic reduction of CD4^+^ T cells and promoting hepatocarcinogenesis [104]. Their further study showed that NASH can impair the effects of M3-RNA vaccine and anti-OX40 antibody treatment against mouse liver tumors induced by intrahepatic injections of B16 melanoma and CT26 colon cancer cells [105]. In contrast, the administration of N-acetylcysteine in NASH mice restored CD4^+^ T cells and resulted in improved effects of the M3-RNA vaccine and anti-OX40 antibody. Moreover, immunotherapy including anti-PD-1 or anti-PD-L1 treatment reduced the overall survival of NASH-HCC patients compared to HCC patients induced with other etiologies [106]. Preclinical mouse model study showed that anti-PD-1 expanded the exhausted and unconventionally activated T cells, such as CXCR6^+^PD-1^+^CD8^+^ T cells, which lost the immune surveillance function and promoted NASH-HCC progression. PD-1^+^CD8^+^ T cells have been reported to be correlated with a poor clinical outcome in HCC patients [107,108]. Thus, the development of NAFLD/NASH modulates intrahepatic immunity to impair the anti-HCC immune response.

## 6. Potential Diagnosis of NAFLD-Related HCC

The development of NAFLD-related HCC is caused by multiple factors. For clinical diagnosis, liver biopsy is still considered the gold standard for clinical decisions. However, it is invasive and may not represent the tumor heterogeneity due to the sample size of the biopsy [109]. In addition, it is not appropriate to be applied to monitor the dynamic progression of HCC. Moreover, the diagnosis of liver fibrosis and cirrhosis may not be helpful in predicting all cases of NAFLD-related HCC, since some patients have developed HCC in the background of NAFLD/NASH without the progression of liver fibrosis and cirrhosis [110,111]. Currently, imaging techniques are essential for the diagnosis of chronic liver disease and HCC. Dynamic multiphase contrast-enhanced computed tomography (CT) scanning and magnetic resonance imaging (MRI) are the most commonly used methods to detect HCC in clinical diagnosis [112,113]. Still, there are some challenges, such as the lack of standardization in image acquisition protocols and optimization of the radiomics analysis procedure [114]. Additional non-invasive or less harmful diagnostic methods, such as biomarkers, score systems, and algorithms, have been investigated to improve the diagnosis of HCC, including its initiation, progression, and potential recurrence. In Table 1, we summarize some developed biomarkers and imaging techniques that provide assistance in the diagnosis of NAFLD-HCC. The combination of different markers and detection methods is helpful in order to provide a precise diagnosis and to make clinical decisions relating to treatment.

## 7. Treatment Options against HCC

Treatment options for HCC can be broadly classified into surgical resection and non-surgical therapies dependent upon the stage of the disease, liver function, availability of donor organs, cost of treatment, and so on [128]. The Westernized diet and sedentary lifestyle promote the progression of NAFLD [129,130]. A cohort study in Europe also showed that physical activity is inversely associated with the risk of HCC [131]. However, excessive exercise can impact the host metabolism to reduce glucose control by impairing mitochondrial function [132]. Here, therapies for regular HCC, NAFLD, and NAFLD-related HCC, including immunotherapy, CAR T cell therapy, peptide treatment, bariatric surgery, and treatment for liver fibrosis, are discussed.

### 7.1. Treatments against HCC

#### 7.1.1. Systemic Therapies and Immunotherapy 

The recurrence of HCC is a big concern after surgical operations. Immunotherapy is helpful to reduce the recurrence of HCC and provides treatment options for advanced HCC that is not suitable for surgical resection. Here, we first briefly summarize some approved first- and second-line treatment options for regular HCC, which may be applied in NAFLD-related HCC treatment.

Sorafenib, a multi-kinase inhibitor, was the first systemic therapy approved by the U.S. Food and Drug Administration (FDA) for patients with unresectable HCC in 2008 [133], and has been approved for the treatment of advanced renal cell carcinoma (RCC) [134]. Lenvatinib is another FDA-approved systemic treatment for unresectable advanced HCC, approved in 2018 [135]. Phase 3 non-inferiority trial data showed that the median survival time for lenvatinib-treated patients was 13.6 months (95% CI, 12.1–14.9 months) compared with a median survival time of 12.3 months (95% CI, 10.4–13.9 months) for patients with sorafenib treatment [135]. In 2020, the FDA approved treatment with atezolizumab in combination with bevacizumab for adult patients with unresectable locally advanced or metastatic HCC without prior systemic therapy [136], since the combined treatment improved OS and progression-free survival compared to treatment with sorafenib [137].

Since 2017, the U.S. FDA has approved several drugs for HCC treatment as the second line after sorafenib treatment, including kinase inhibitors (regorafenib, lenvatinib, cabozantinib, and ramucirumab), immune checkpoint inhibitors (nivolumab and pembrolizumab), and monoclonal antibodies (atezolizumab plus bevacizumab). In 2017, regorafenib was approved as the first drug by the FDA for the treatment of advanced HCC in patients who had previously been treated with sorafenib [138]. In 2018, pembrolizumab, a monoclonal antibody against PD-1, was approved by the FDA for the treatment of patients with HCC who have been previously treated with sorafenib. In 2019, the FDA approved another drug, cabozantinib, for patients with HCC with prior treatment with sorafenib, since phase 3 trial results indicated that cabozantinib treatment resulted in longer overall survival and progression-free survival than a placebo in previously treated patients with advanced HCC [139]. Following up, the FDA approved ramucirumab as a sole treatment for HCC patients who have serum AFP levels ≥ 400 ng/mL with prior treatment with sorafenib. In addition, there are some combined treatments, such as nivolumab and ipilimumab, that may improve outcomes [140]. 

Additionally, there are increasing numbers of clinical trials evaluating different therapies in a variety of combinations for the systemic treatment of HCC at different disease stages with the Barcelona Clinic for Liver Cancer (BCLC) criteria, which is well summarized in another review [141]. The FDA-approved treatments for HCC are summarized in Table 2.

#### 7.1.2. CAR T Cells

Chimeric antigen receptor (CAR) T cells have been tested in a variety of diseases, including aging [142], autoimmune disease [143], and tumors such as B-cell acute lymphatic leukemia [144] and myeloma [145]. The principle of CAR T cell immunotherapy is to engineer T cells to express CARs, which consist of an extracellular antigen recognition domain fused to intracellular T-cell receptor (TCR) signaling and co-stimulatory domains [146]. Genetically engineered CAR T cells recognize antigens on malignancy cells to effectively damage them and overcome tolerance. A phase 1 trial showed that CAR-glypican-3 (GPC-3) T-cell therapy showed some early signs of effectiveness against advanced HCC in patients [147], such as decreased counts of different lymphocytes. In addition, there are other tumor-targeting antigens against HCC, such as AFP [148] and New York esophageal squamous cell carcinoma-1 (NY-ESO-1) [120]. In addition, these antigens including *AFP* and *GPC-3* are observed in NAFLD-related HCC [149].

#### 7.1.3. Peptides

Peptide vaccination has been applied in the clinic to treat HCC. In a study of vaccination with human telomerase reverse transcriptase-derived peptide 461 (hTERT461, VYGFVRACL) in a total of 14 HCC patients, 11 patients (71.4%) showed hTERT461-specific cytotoxic T cells (CTLs) in blood post-vaccination at 4 weeks [161]. Of the response patients, 57.1% of patients (five of them) did not show HCC recurrence. In contrast, HCC recurred in all three patients (100.0%) without hTERT461-specific CTLs. In 15 HCC patients after vaccination with AFP-derived peptide 357 (AFP357) vaccine, 53.3% of patients showed slow tumor growth and 26.7% had AFP357-specific CD8 T cells [162]. Only one patient had a complete response more than 2 years; the functional T cells in this patient expressed a high avidity for AFP-specific T-cell receptors. Anti-cancer peptides are also potential treatment options for liver cancer [163].

### 7.2. Treatment against NAFLD

Bariatric surgery (BS) or weight loss surgery has been well established to provide excellent weight loss outcomes and improvements in comorbid medical conditions, including diabetes [164], NAFLD [165], cardiac function [166], and cancer [167]. Multiple mechanisms are involved in the effectiveness of weight loss surgery, including restriction (sleeve gastrectomy and gastric bypass) and malabsorption (gastric bypass and biliopancreatic diversion with duodenal switch) and through gut enteroendocrine hormonal effects [168] (sleeve gastrectomy, gastric bypass, and biliopancreatic diversion with duodenal switch). Weight loss surgery alters the gut microbiota, as well as circulating bile acids [169] and many blood metabolites [170]. These mechanisms are likely intricately related and function to regulate the effects of weight loss surgery on a person’s health [169]. However, there is a lack of evidence for the treatment of NAFLD-related HCC in clinical trials.

Many treatment agents have been tested in clinical trials for the treatment of NAFLD or NASH with promising effects, including polyphenols, bile acids, diet intervention, herb medicines, anti-inflammatory or antioxidant agents, hormones, and symbiotics (Table 3). 

### 7.3. Treatment against Liver Fibrosis

*TGF-β* is a predominant profibrotic gene that causes the activation of hepatic stellate cells (HSCs) in the liver independently of causal factors such as a high-fat diet, alcohol, and other toxins such as carbon tetrachloride (CCL_4_). Strategies that block the *TGF-β* signaling pathway can inhibit the progression of liver fibrosis [181]. Bone morphogenetic proteins (BMPs) belonging to the large TGF-β family play an important role in tissue homeostasis. Accumulating evidence indicates that BMPs are involved in the development and progression of liver fibrosis and liver regeneration [182,183], becoming a new target for liver fibrosis. In addition, some other drugs can ameliorate NAFLD/NASH-associated liver fibrosis, such as the farnesoid X receptor (FXR) agonists obeticholic acid and isotschimgine [184,185]. Furthermore, changing lifestyles, such as the consumption of a healthy diet and appropriate exercise, also can prevent fibrosis progression in NAFLD patients.

**Figure 3:**
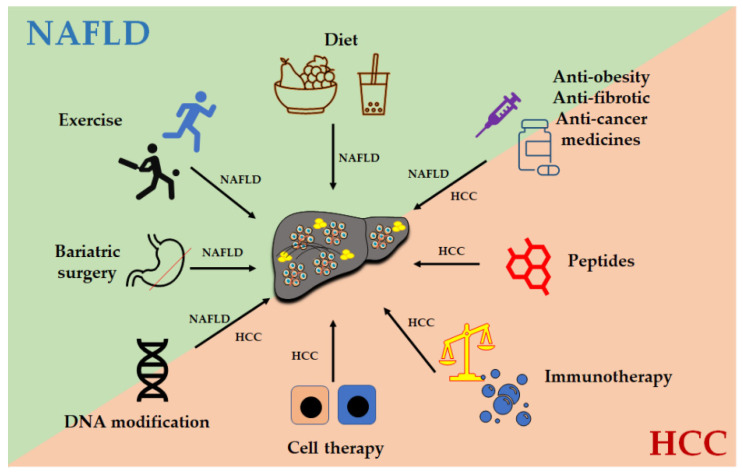
Treatment options for NAFLD-related HCC. Abbreviations: NAFLD: nonalcoholic fatty liver disease; NASH: nonalcoholic steatohepatitis; HCC: hepatocellular carcinoma.

Overall, the treatment options (Figure 3) for NAFLD-related HCC, targeting liver fibrosis, lipid accumulation, cancer cells, and immune responses, can be selected according to the pathogenesis of the liver disease and the health condition of the patient.

## 8. Conclusions

The prevalence of NAFLD-related HCC is increasing in developed countries and developing countries due to the consumption of fast food or a Western-like diet, less physical exercise, and an increased prevalence of obesity and diabetes. Patients with advanced NAFLD or NASH and liver fibrosis progression are at a higher risk of developing HCC. Imaging is the most commonly used technique for HCC diagnosis. The development of other non-invasive diagnostic methods is also critically important for better clinical treatment and prevention of HCC. Combined diagnosis with multiple diagnostic methods provides higher sensitivity and specificity for monitoring NAFLD-related HCC. Although there are several FDA-approved drug treatments, the overall survival rate and survival time are still not promising. In addition, some treatments such as bariatric surgery are more beneficial at the early stage of NAFLD but show no evidence of helping HCC therapy in clinical trials. With the development of large databases, along with artificial intelligence and machine learning, precision medicine in the future will improve the diagnosis of NAFLD-related HCC and provide better options for personal precise treatment of the disease.

## Figures and Tables

**Table 1:** The potential HCC diagnostic methods.

Methods	Target	Disease	References
Imaging techniques ^1^	Various data in clinical records, including images from abdominal ultrasonography, computed tomography, magnetic resonance imaging, electronic health records, liver pathology, data from wearable devices, and multi-omics measurements can be used to predict liver fibrosis, cirrhosis, NAFLD, and for the differentiation of benign tumors from HCC.	NAFLD, liver fibrosis, cirrhosis, and HCC	[115,116,117]
Polygenic risk scores (PRS)	Variants in *PNPLA3*-*TM6SF2*-*GCKR*-*MBOAT7* are combined in a hepatic fat PRS (PRS-HFC), and then adjusted for HSD17B13 (PRS-5). Similarly, a genetic risk score (GRS) based on three genetic variants of *PNPLA3*-*TM6SF2*-*HSD17B13* is applied to evaluate how fatty liver disease influences the risk of cirrhosis and HCC.	NAFLD-related HCC	[118,119]
Biomarkers	For example, serum levels of inter-alpha-trypsin inhibitor heavy chain 4 are significantly increased in HCC-NAFLD patients compared to those in patients with simple steatosis, NASH, and virus-related HCC. There are some other biomarkers, such as miRNAs, lncRNAs, and circulating tumor DNA (ctDNA).	NAFLD-related HCC	[109,120,121,122,123]
Biomarker-based diagnostic algorithm (GALAD)	A GALAD score based on gender, age, and biomarker alpha-fetoprotein (AFP), AFP-L3, and Des-gamma-carboxy prothrombin (DCP).	Early-stage of HCC	[124,125,126]
Epigenetic factors with or without combined biomarkers	Epigenetic factors such as circular RNA *SMARCA5* can be used as potential new biomarkers for hepatocellular carcinoma. In combination with serum lncRNA *linc00152, UCA1* and *AFP* can show better predictive ability, with areas under the curve (AUC) of 0.912% and 82.9% sensitivity and 88.2% specificity.	Hepatitis, cirrhosis, and HCC	[122,127]

^1^ Imaging techniques, especially dynamic multiphase contrast-enhanced computed tomography (CT) scanning and magnetic resonance imaging (MRI), are the most commonly used methods for the clinical diagnosis of HCC.

**Table 2:** FDA-approved treatments for HCC.

Approval Year	Selection	Treatment	Targets	References
2008	First-line	Sorafenib	Vascular endothelial growth factor receptor (VEGFR)-2 and -3, platelet-derived growth factor receptor (PDGFRβ), receptor tyrosine kinases RET and KIT, and Raf kinase	[150,151]
2017	Second-line	Regorafenib	VEGFR1-3, TEK receptor tyrosine kinase or angiopoietin-1 receptor (TIE2), fibroblast growth factor receptor 1 (FGFR1) and PDGFRβ, oncogenic kinases c-KIT, RET, and c-RAF/RAF-1	[138,152]
2017	Second-line	Nivolumab	PD-1	[153]
2018	First-line	Lenvatinib	An oral inhibitor of VEGFR 1-3, FGFR 1-4, PDGFRα, receptor tyrosine kinases RET and KIT	[135,154]
2018	Second-line	Pembrolizumab	PD-1	[155]
2019	Second-line	Cabozantinib	Tyrosine kinases, including MET, AXL, and VEGFR 1-3	[139,156]
2019	Second-line	Ramucirumab	VEGFR2	[157]
2020	Second-line	Nivolumab and Ipilimumab	PD-1 and CTLA-4	[158,159]
2020	First-line	Atezolizumab plus Bevacizumab	PD-L1 and VEGF	[136,160]

**Table 3:** The completed clinical trials for NAFLD/NASH treatment.

Liver Disease	Treatment	Class	Effect	Trial	References
NAFLD	Curcumin	Polyphenol	Daily supplementation of curcumin (250 mg) for 2 months caused a significant reduction in hepatic steatosis and enzymes in patients with NAFLD compared to placebo.	A double-blind, randomized, placebo-controlled trial.	[171]
NAFLD	Ursodeoxycholic acid (UDCA)	Bile acid	Treatment with UDCA (15 mg/kg/d) for 3 months, the level of enzymes alanine aminotransferase (ALT), aspartate transaminase (AST), and glutamyltransferase decreased. After 6-month treatment, body weight, fatty liver index, total cholesterol, low-density lipoprotein, and triglyceride were significantly reduced.	An open-label, multicenter, international noncomparative trial.	[172]
NAFLD with T2DM	Tofogliflozin	A selective sodium-glucose cotransporter 2 inhibitor	Oral treatment with 20 mg tofogliflozin or 15–30 mg daily for 24 weeks significantly decreased body weight and hepatic steatosis.	A randomized prospective open-label controlled trial.	[173]
NAFLD	Diet and activity	Physical activity (PA), a low glycemic index Mediterranean diet (LGIMD), or their combined effect.	After 45 days, there was a statistically significant reduction in the NAFLD score in each group except for the control diet-treated group. After 90 days, the combined treatment showed the best effect on the reduction of NAFLD score.	A randomized clinical trial.	[174]
NAFLD	Sumac	Herbal medicine	After a 12-week intervention, the sumac-treated group displayed a greater reduction in hepatic fibrosis and enzymes ALT and AST, fasting blood sugar, serum insulin, and HOMA-IR (insulin resistance index), as well as higher QUICKI (insulin sensitivity index) compared to the placebo.	A randomized controlled trial.	[175]
NAFLD	Propolis	An anti-inflammatory agent	Patients with propolis at a dose of 250 mg twice daily for 4 months compared to placebo showed a significant improvement in hepatic steatosis and a significant reduction of liver stiffness, as well as serum high-sensitivity C-reactive protein (hs-CRP).	A randomized clinical trial.	[176]
NAFLD	Melatonin	A hormone	Administration of 6 mg/day melatonin significantly reduced body weight, waist and abdominal circumference systolic and diastolic blood pressure, serum levels of leptin, ALT, AST, and hs-CRP, and the grade of fatty liver compared with the placebo.	A randomized double-blind clinical trial.	[177]
NAFLD	Vitamin E	An antioxidant vitamin	Treatment with vitamin E improved liver injury and steatosis.	A single-center prospective trial.	[178]
NASH	Cenicriviroc	A dual antagonist C-C chemokine receptor type 2 (CCR2) and CCR5	Therapy with cenicriviroc showed an antifibrotic effect without impacting steatohepatitis at year 1 in responders, which was maintained in year 2 with a greater effect in advanced fibrosis.	A randomized, controlled study.	[179]
NAFLD	Synbiotic	A mixture of probiotics and prebiotics	Administration of a synbiotic combination of probiotic and prebiotic for one year changed gut microbiota but did not reduce liver fat content or markers of liver fibrosis.	A double-blind phase 2 trial.	[180]

## Data Availability

All the data supporting the reported results can be found in the references.
